# Supplementary material for: Comparative Analysis of the Effect of Gamma-, Electron, and Proton Irradiation on Transcriptomic Profile of Hordeum vulgare L. Seedlings: In Search for Molecular Contributors to Abiotic Stress Resilience
Source: Plants (Basel). 2024 Jan 23;13(3):342. doi: 10.3390/plants13030342 (PMC10857502; doi:10.3390/plants13030342)
Supplement: Supplementary file 1 [file plants-13-00342-s001.zip › Figures_S1-S17_GO_terms visualization.pdf]

Legend

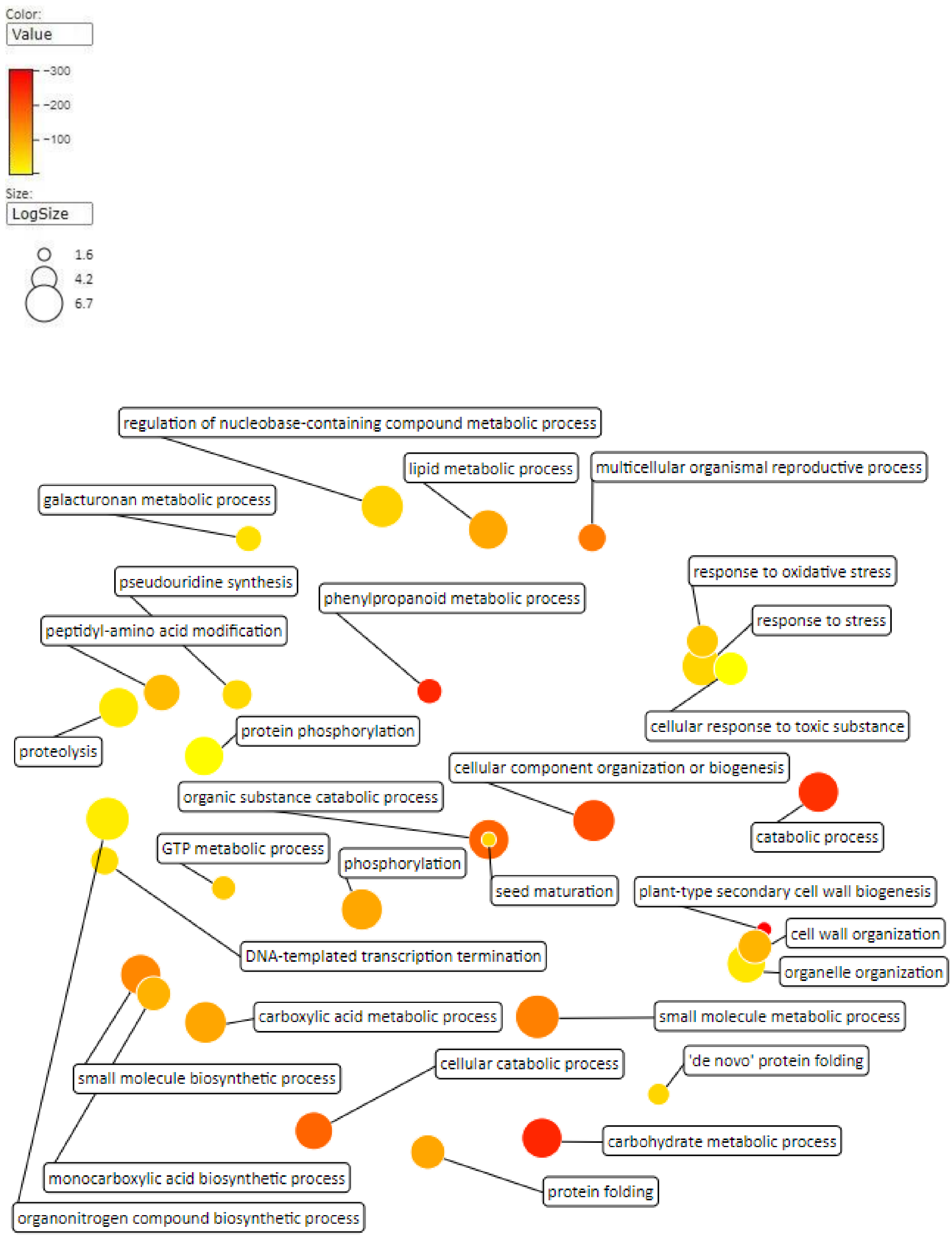

Figure S1. Scheme of the most significantly enriched GO terms for upregulated DEGs shared between γ-radiation and electron beam in terms of Biological Process, visualized by REVIGO.

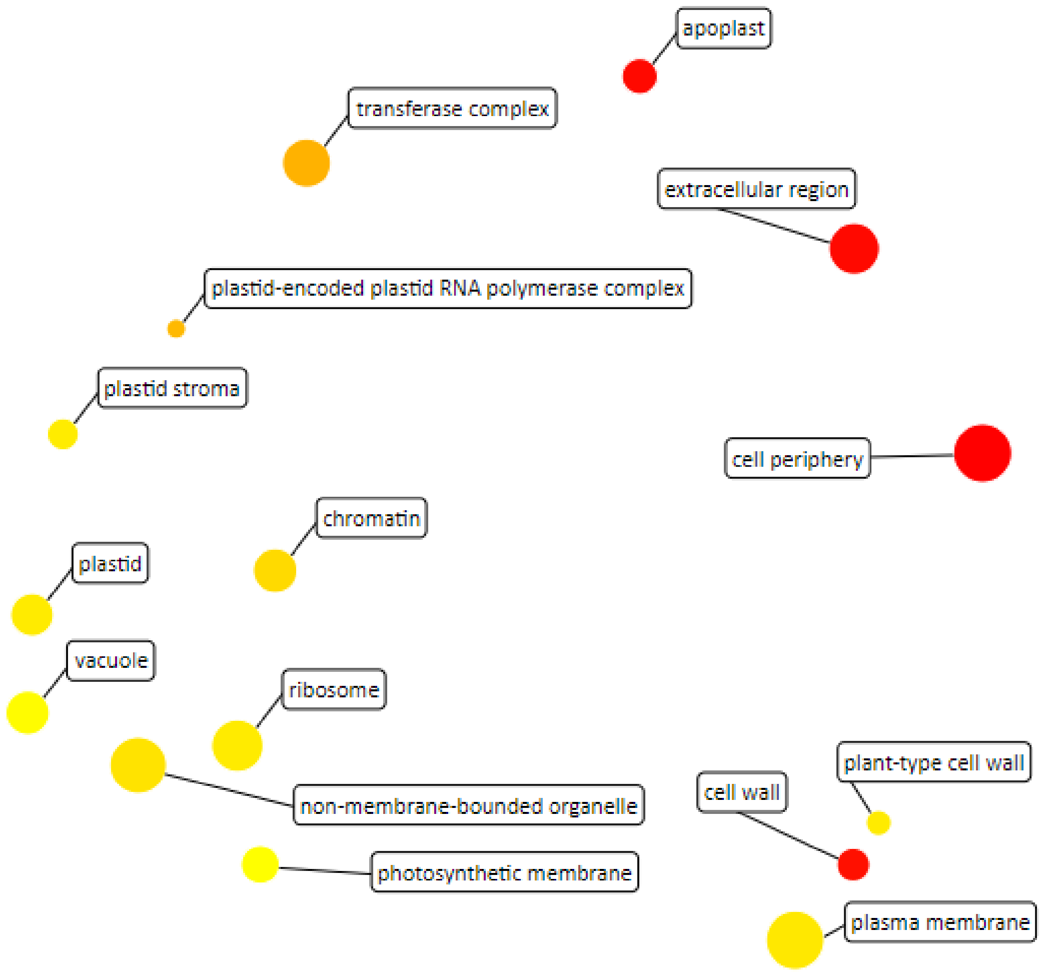

Figure S2. Scheme of the most significantly enriched GO terms for upregulated DEGs shared between γ-radiation and electron beam in terms of Cellular Component, visualized by REVIGO.

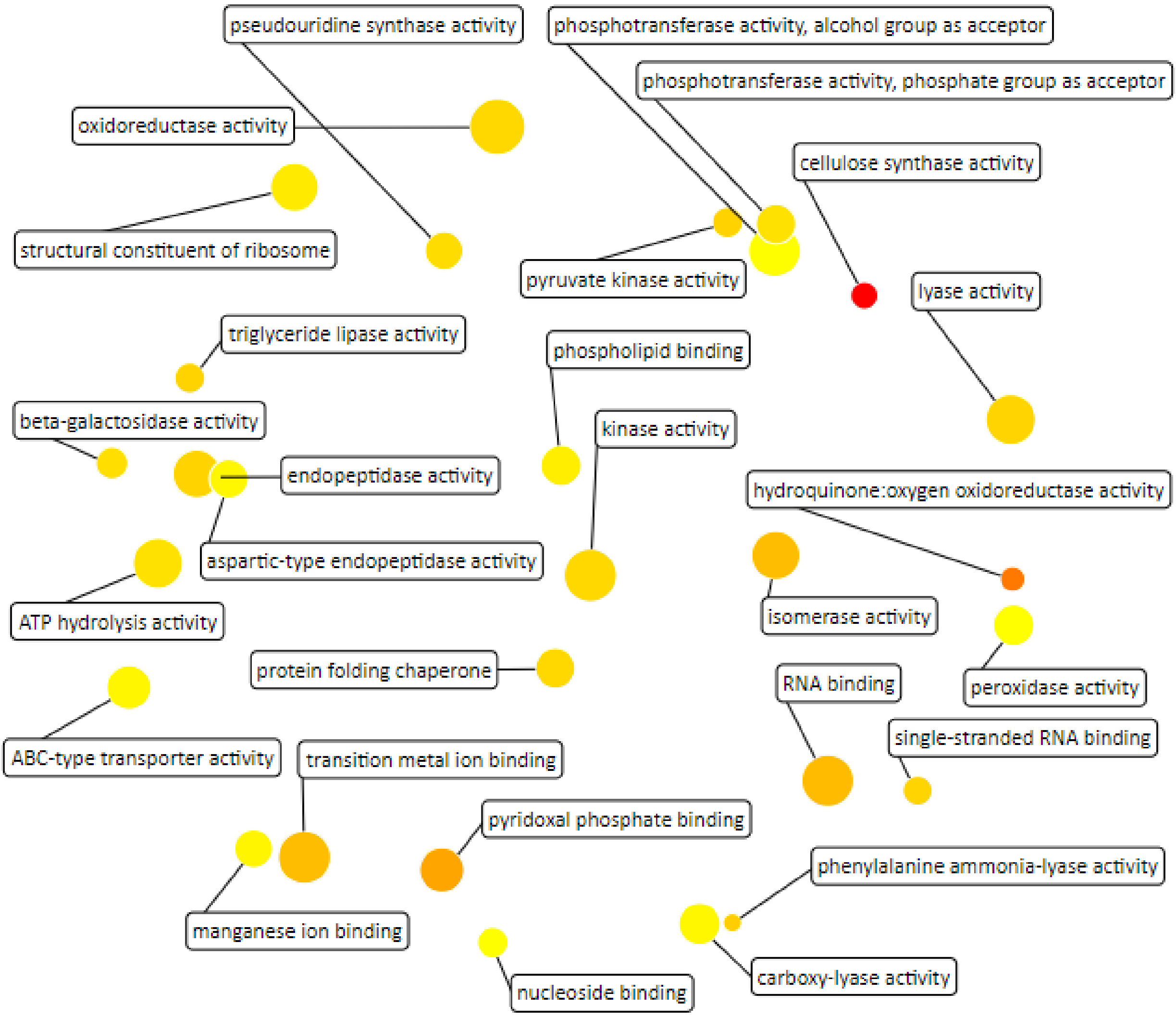

Figure S3. Scheme of the most significantly enriched GO terms for upregulated DEGs shared between γ-radiation and electron beam in terms of Molecular Function, visualized by REVIGO.

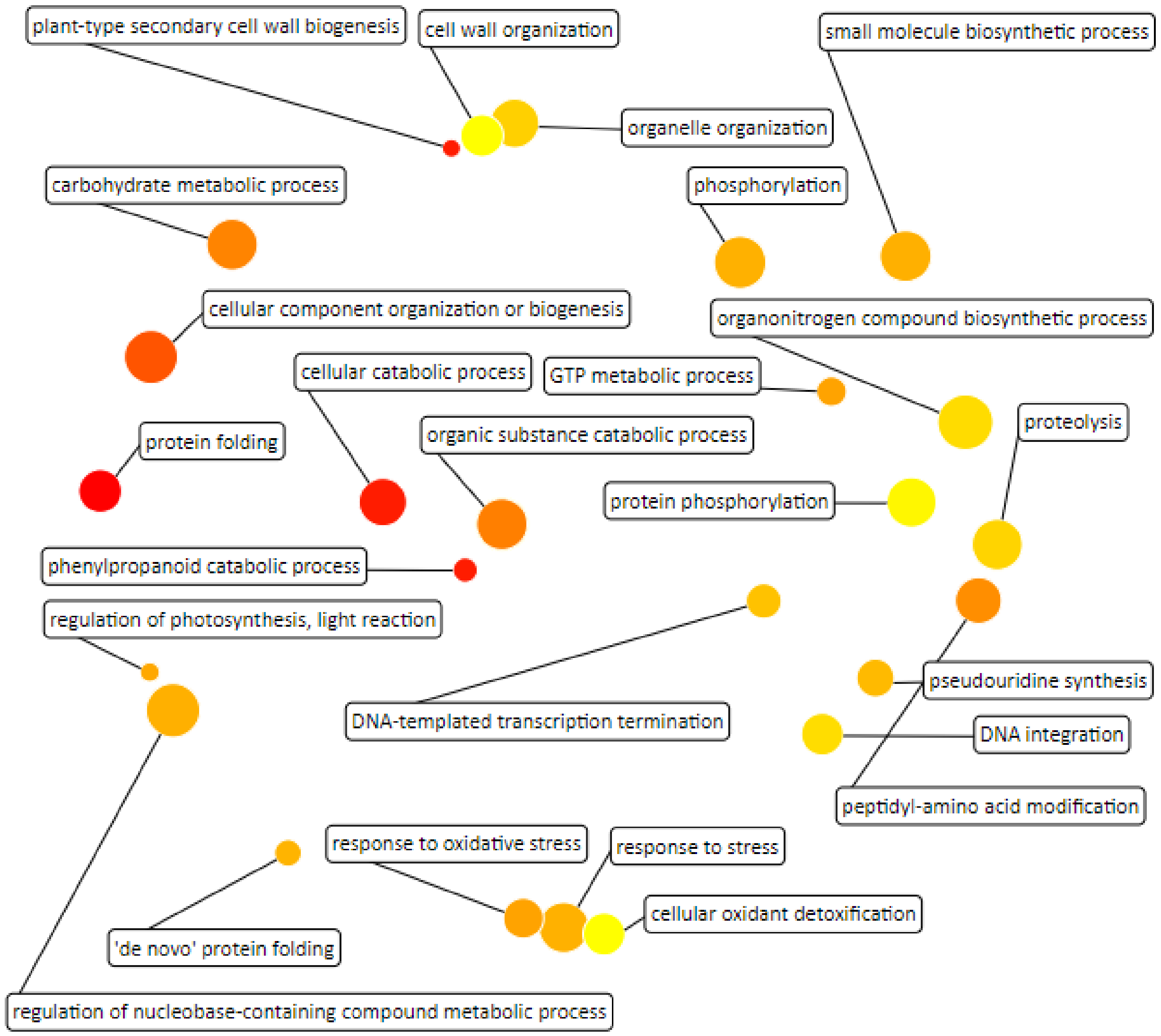

Figure S4. Scheme of the most significantly enriched GO terms for upregulated DEGs shared between γ-radiation and proton beam in terms of Biological Process, visualized by REVIGO.

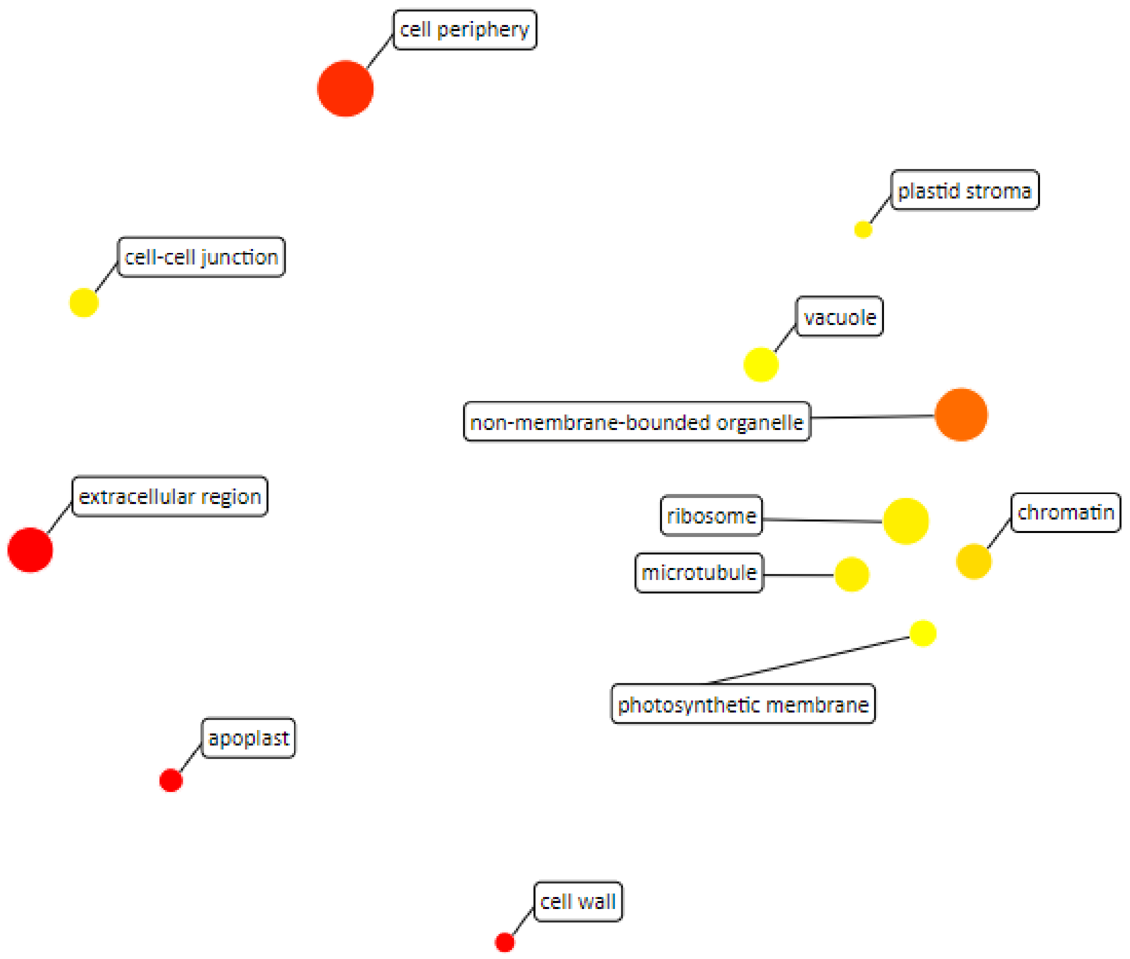

Figure S5. Scheme of the most significantly enriched GO terms for upregulated DEGs shared between γ-radiation and proton beam in terms of Cellular Component, visualized by REVIGO.

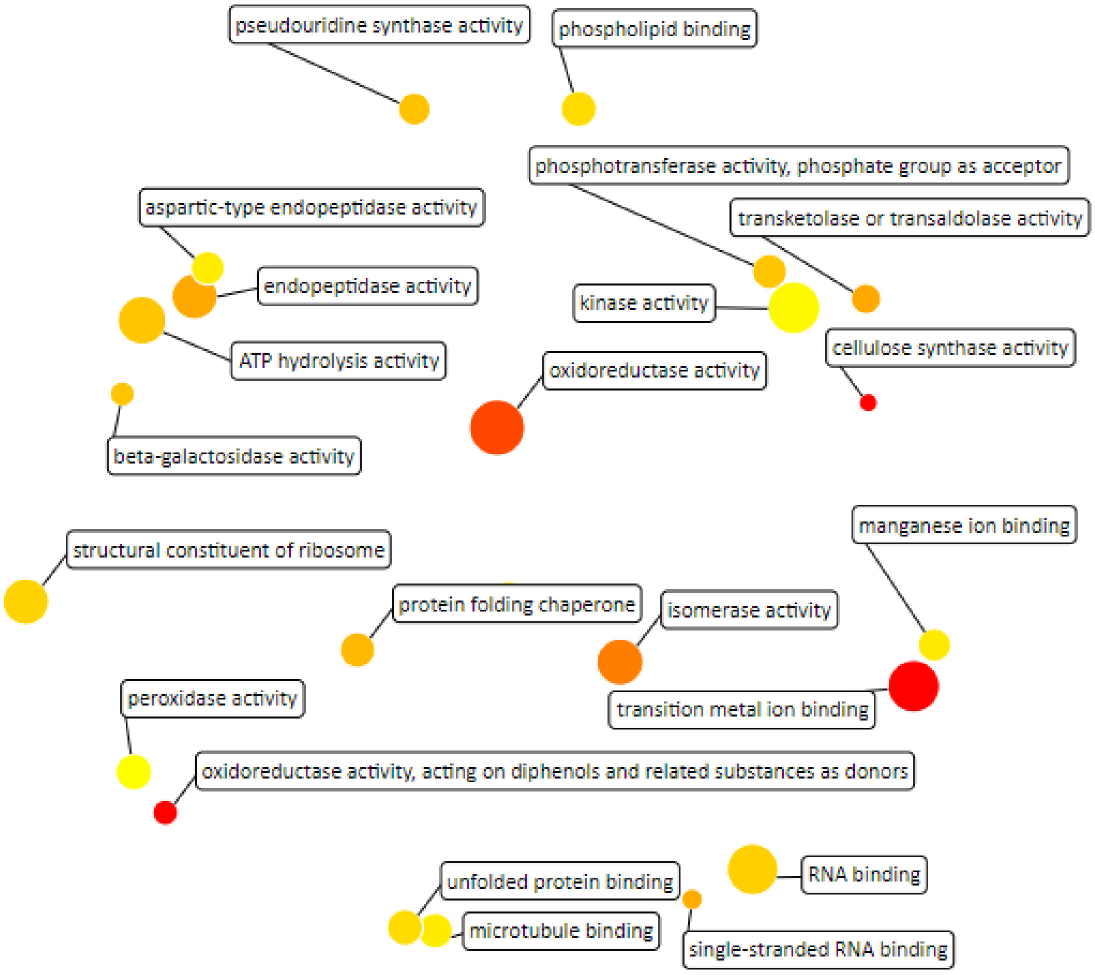

Figure S6. Scheme of the most significantly enriched GO terms for upregulated DEGs shared between γ-radiation and proton beam in terms of Molecular Function, visualized by REVIGO.

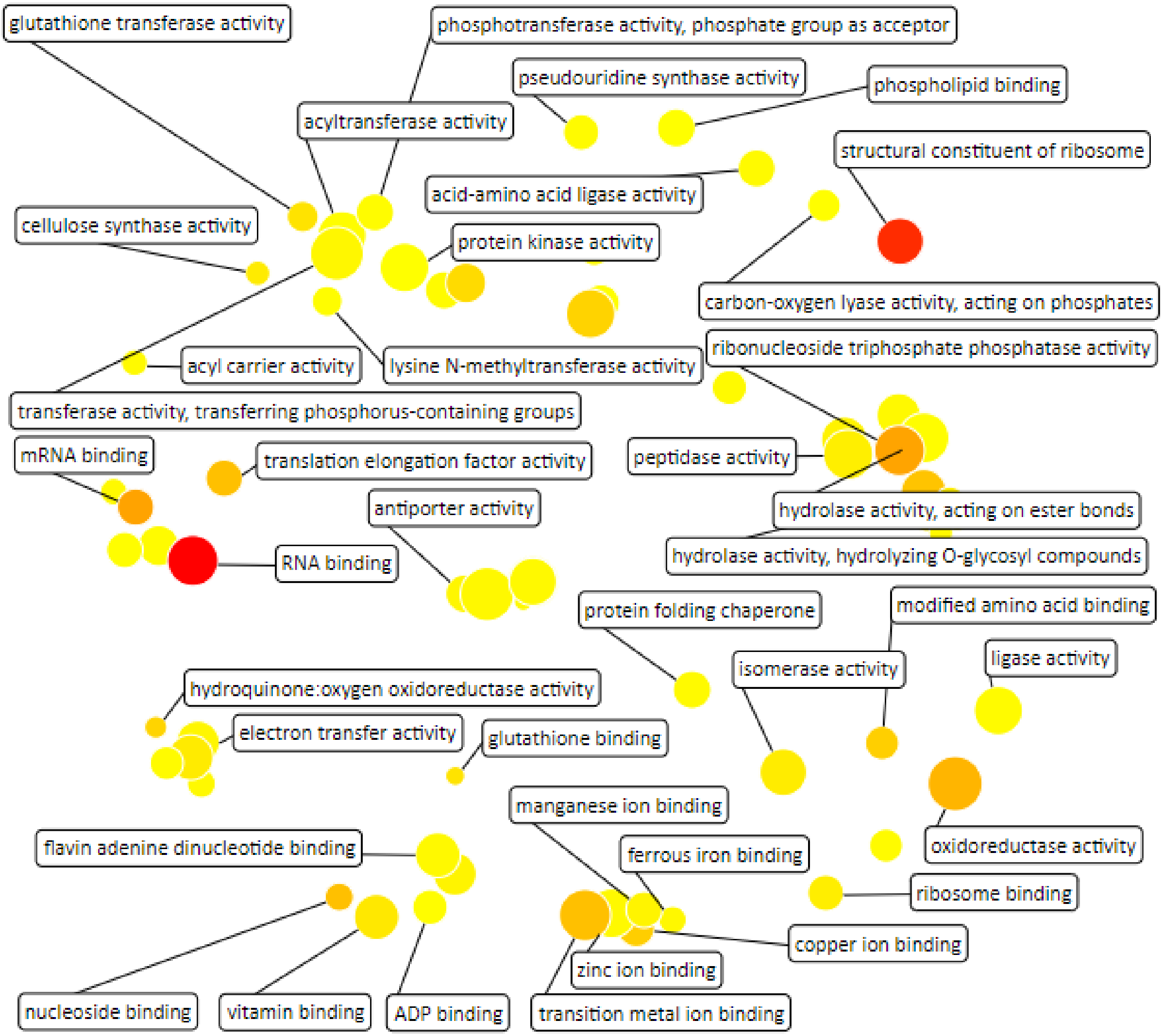

Figure S7. Scheme of the most significantly enriched GO terms for upregulated DEGs shared between electron and proton beam in terms of Molecular Function, visualized by REVIGO.

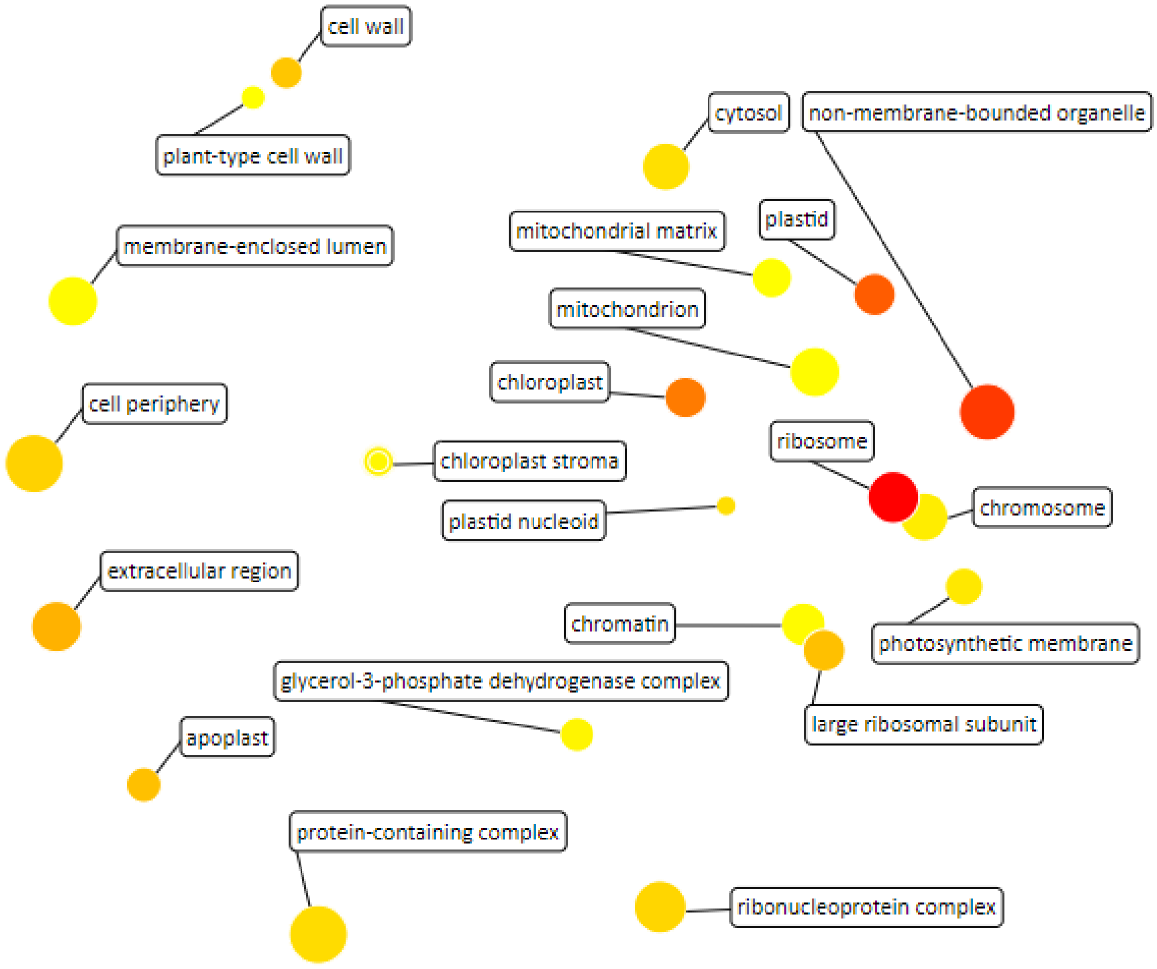

Figure S8. Scheme of the most significantly enriched GO terms for upregulated DEGs shared between electron and proton beam in terms of Cellular Component, visualized by REVIGO.

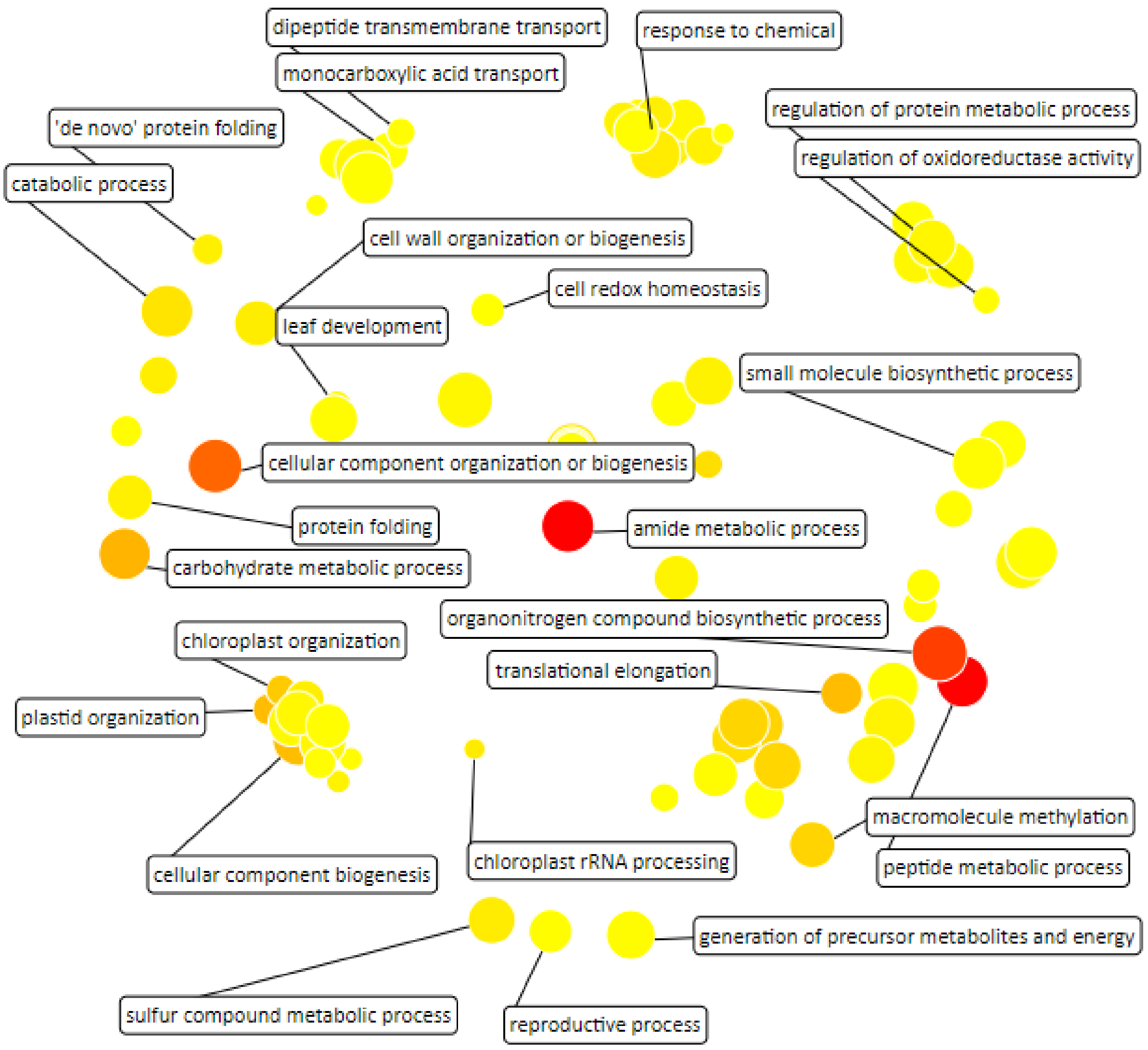

Figure S9. Scheme of the most significantly enriched GO terms for upregulated DEGs shared between electron and proton beam in terms of Biological Process, visualized by REVIGO.

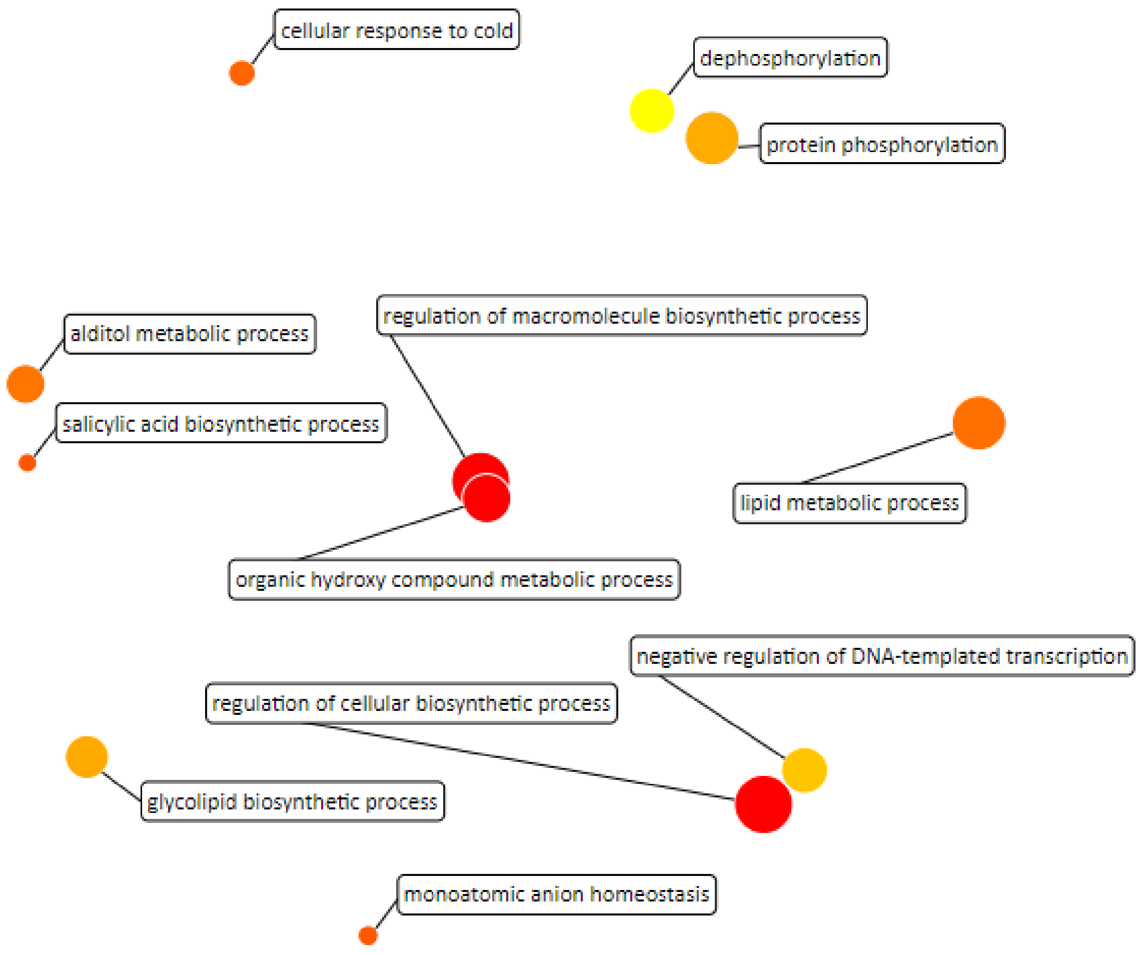

Figure S10. Scheme of the most significantly enriched GO terms for downregulated DEGs shared between electron and proton beam in terms of Biological Process, visualized by REVIGO.

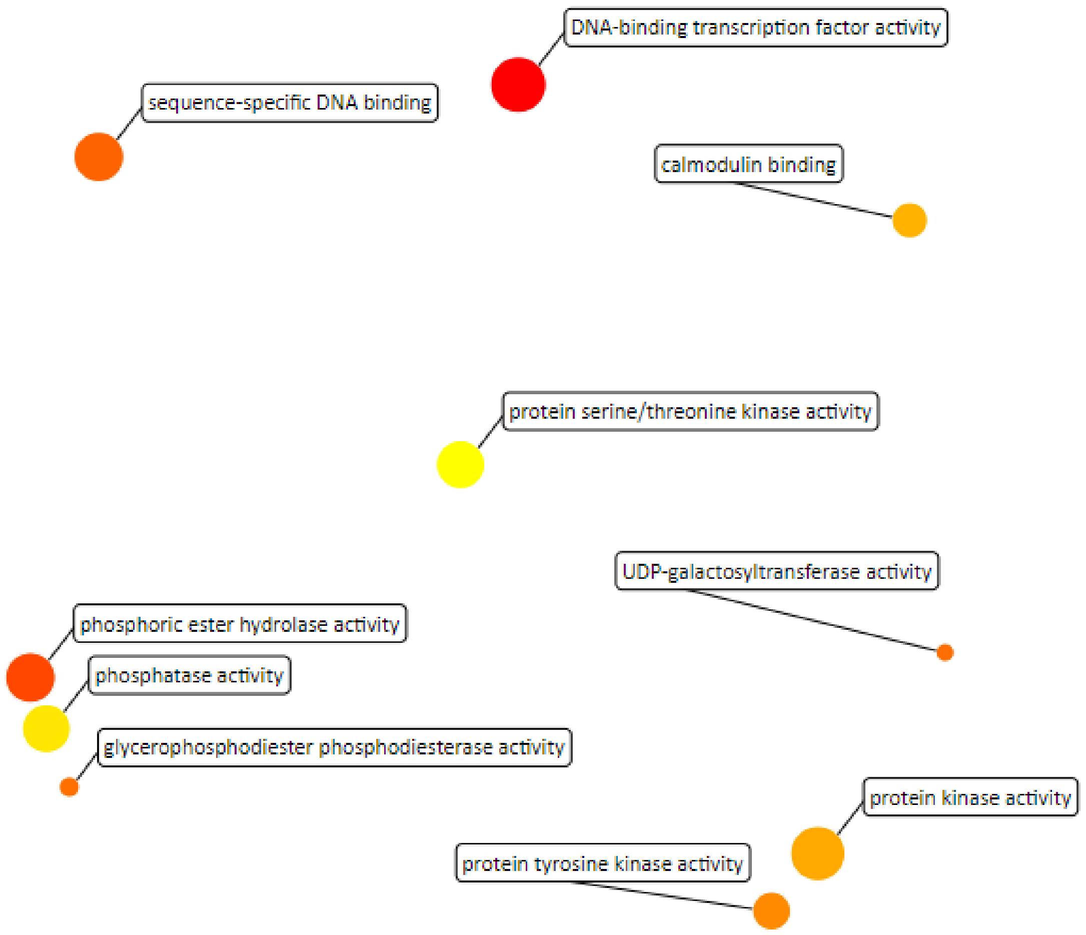

Figure S11. Scheme of the most significantly enriched GO terms for downgulated DEGs shared between electron and proton beam in terms of Molecular Function, visualized by REVIGO.

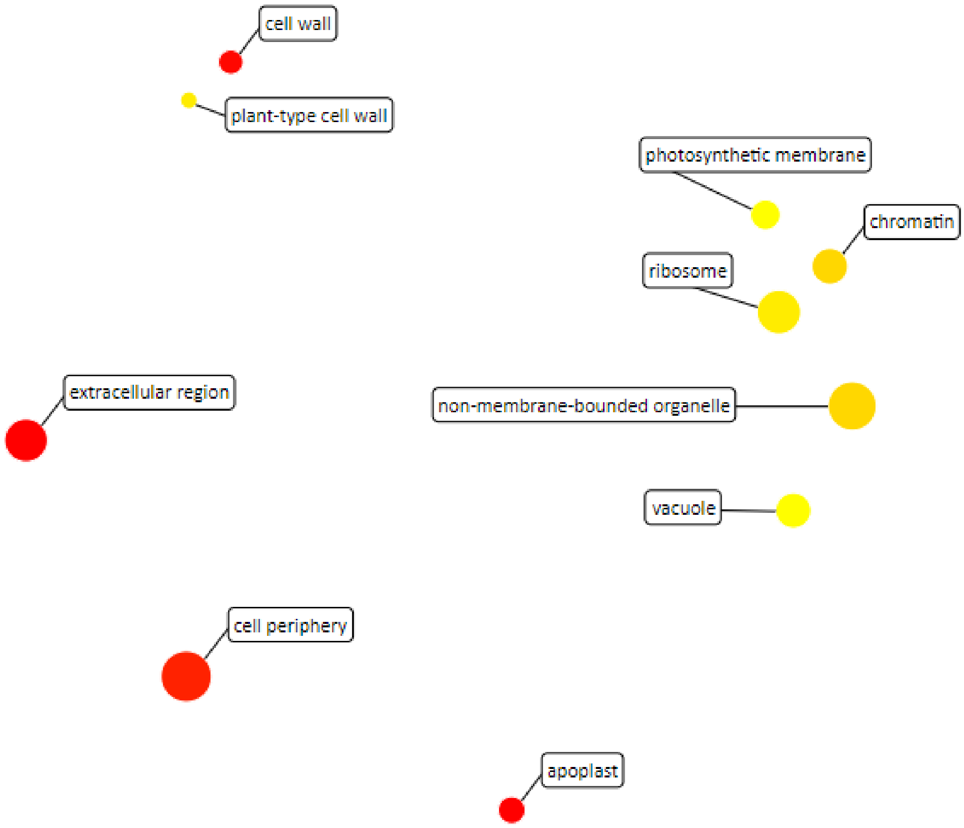

Figure S12. Scheme of the most significantly enriched GO terms shared among all types of radiation applied in terms of Cellular Component, visualized by REVIGO.

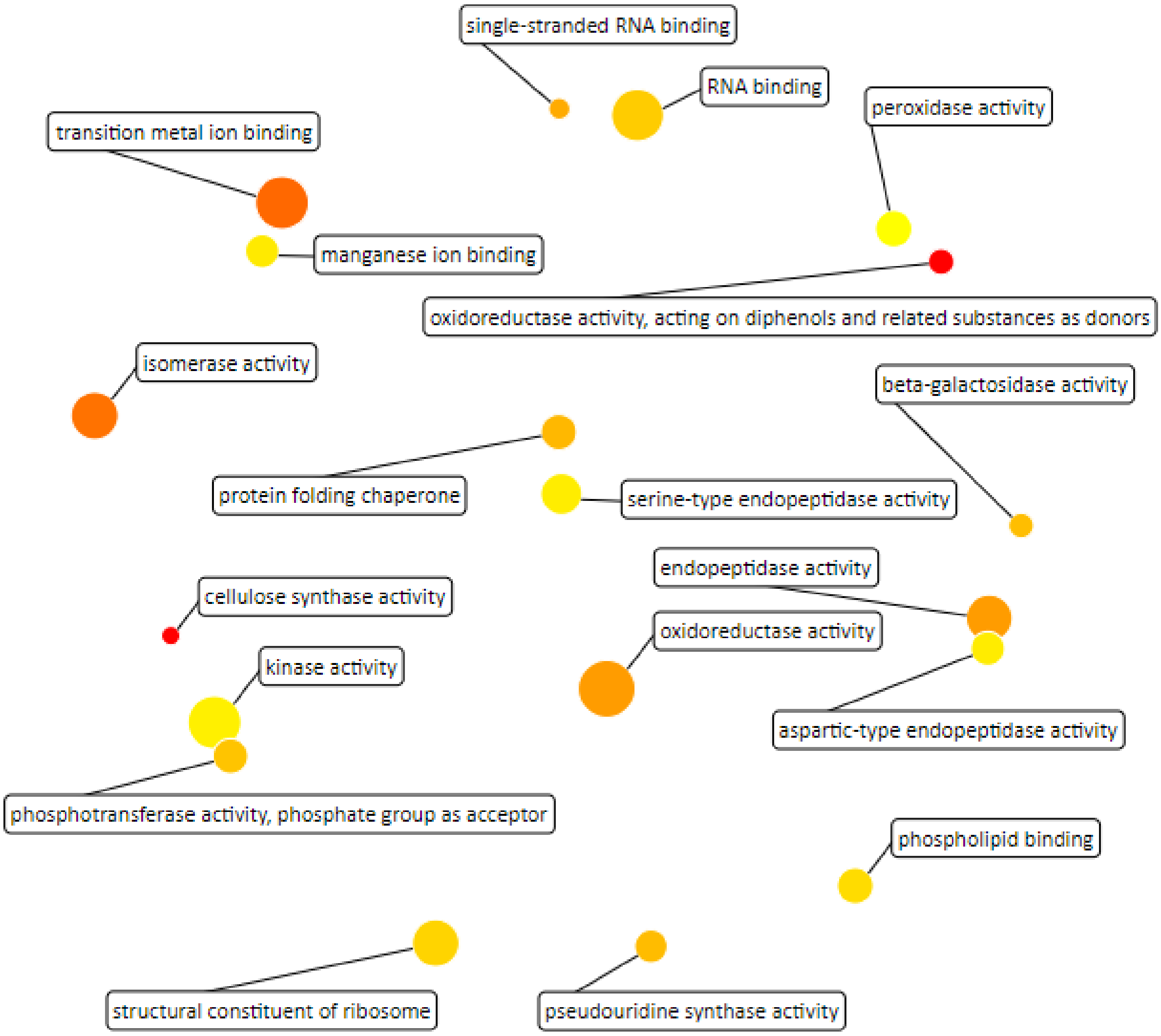

Figure S13. Scheme of the most significantly enriched GO terms shared among all types of radiation applied in terms of Molecular Function, visualized by REVIGO.

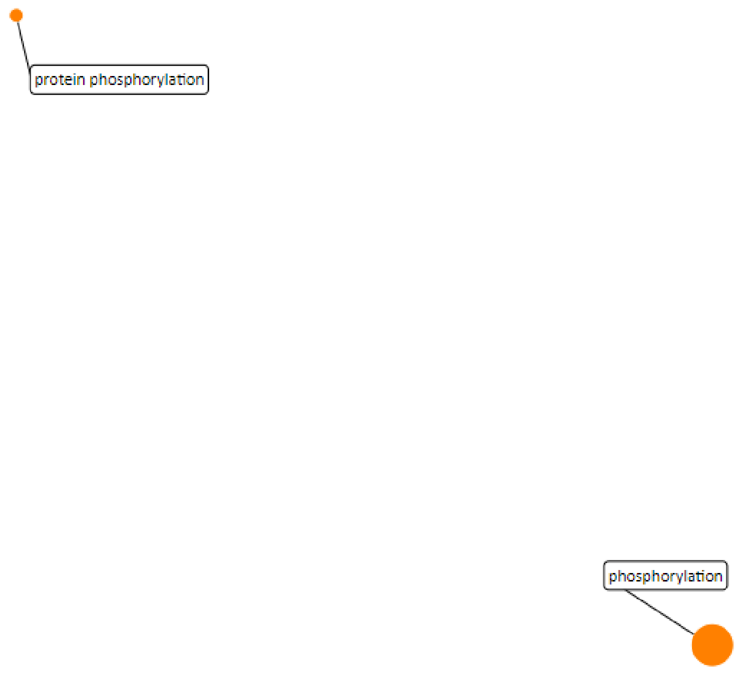

Figure S14. Scheme of the most significantly enriched GO terms for downregulated DEGs shared between γ-radiation and electron beam in terms of Biological Process, visualized by REVIGO.

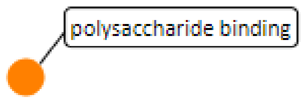

Figure S15. Scheme of the most significantly enriched GO terms for downregulated DEGs shared between γ-radiation and electron beam in terms of Molecular Function, visualized by REVIGO.

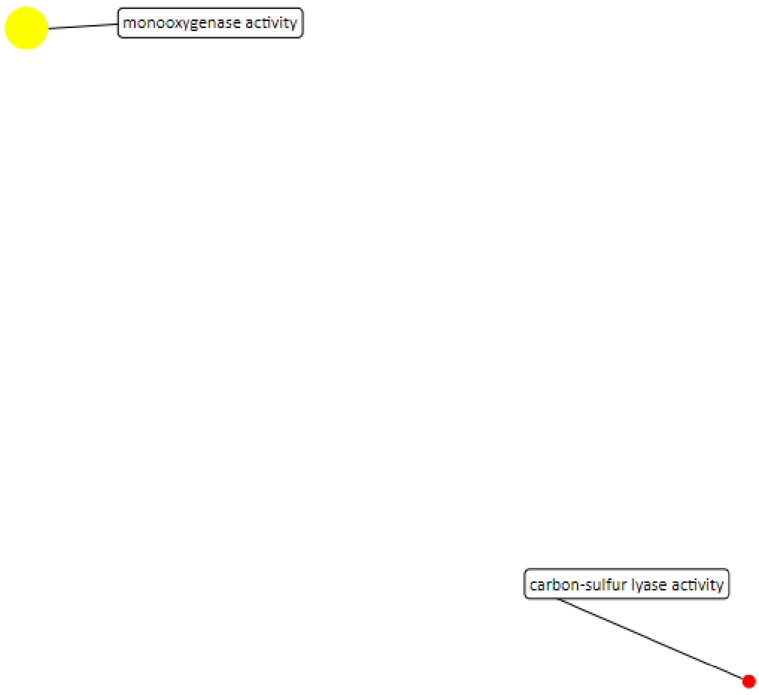

Figure S16. Scheme of the most significantly enriched GO terms for downregulated DEGs shared between γ-radiation and proton beam in terms of Molecular Function, visualized by REVIGO.

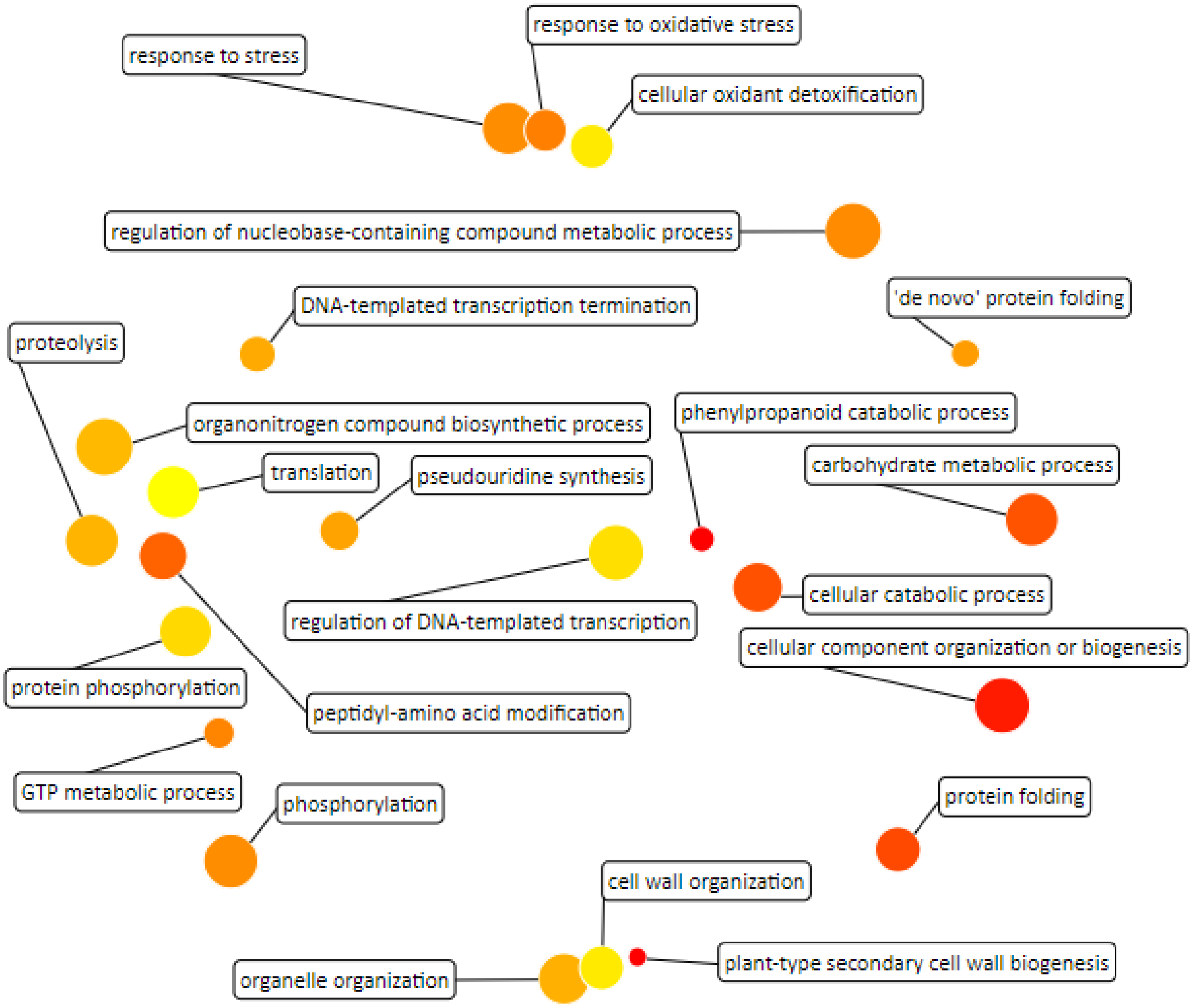

Figure S17. Scheme of the most significantly enriched GO terms shared among all types of radiation applied in terms of Biological Process, visualized by REVIGO.
